# Underlying Mechanisms of Gene–Environment Interactions in Externalizing Behavior: A Systematic Review and Search for Theoretical Mechanisms

**DOI:** 10.1007/s10567-015-0196-4

**Published:** 2015-11-04

**Authors:** Joyce Weeland, Geertjan Overbeek, Bram Orobio de Castro, Walter Matthys

**Affiliations:** Utrecht Centre for Child and Adolescent Studies, Utrecht University, PO Box 15.804, 1001 NH Amsterdam, The Netherlands; Department of Child and Adolescent Studies, Utrecht University, Utrecht, The Netherlands; Research Institute of Child Development and Education, University of Amsterdam, Amsterdam, The Netherlands

**Keywords:** Review, Gene–environment interactions, Externalizing behaviors, Postnatal family adversity, Theoretical mechanisms

## Abstract

Over the last decade, several candidate genes (i.e., *MAOA*, *DRD4*, *DRD2*, *DAT1*, *5*-*HTTLPR*, and *COMT*) have been extensively studied as potential moderators of the detrimental effects of postnatal family adversity on child externalizing behaviors, such as aggression and conduct disorder. Many studies on such candidate gene by environment interactions (i.e., cG × E) have been published, and the first part of this paper offers a systematic review and integration of their findings (*n* = 53). The overview shows a set of heterogeneous findings. However, because of large differences between studies in terms of sample composition, conceptualizations, and power, it is difficult to determine if different findings indeed illustrate inconsistent cG × E findings or if findings are simply incomparable. In the second part of the paper, therefore, we argue that one way to help resolve this problem is the development of theory-driven a priori hypotheses on which biopsychosocial mechanisms might underlie cG × E. Such a theoretically based approach can help us specify our research strategies, create more comparable findings, and help us interpret different findings between studies. In accordance, we describe three possible explanatory mechanisms, based on extant literature on the concepts of (1) emotional reactivity, (2) reward sensitivity, and (3) punishment sensitivity. For each mechanism, we discuss the link between the putative mechanism and externalizing behaviors, the genetic polymorphism, and family adversity. Possible research strategies to test these mechanisms, and implications for interventions, are discussed.

## Introduction

Caspi et al. (2002) found the adverse effect of maltreatment on antisocial behaviors to be moderated by a functional polymorphism in the monoamine oxidase A (*MAOA*) gene. Following this exciting discovery, a fast paced research field emerged focusing on candidate gene by environment interactions (cG × E). Since then the original findings have been replicated, as well as extended to interactions between a broad variety of candidate genes and environmental risk factors in predicting different forms of externalizing behaviors. These findings have taught us much about the interplay of genes and environment in the development of externalizing behaviors. However, the literature has also raised some criticism and important growing pains of the field are difficulties with replication and contradictory findings, which complicates creating a consistent picture (e.g., Dick et al. 2015; Duncan and Keller 2011; Jaffee et al. 2013; Rutter 2012). Multiple meta-analyses on cG × E in externalizing behaviors have already been published, but these have mostly focused on the *MAOA* gene (Byrd and Manuck 2014; Kim-Cohen et al. 2006; Taylor and Kim-Cohen 2007). A complete overview of cG × E in externalizing behaviors is missing (for a general overview of cG × E in psychopathology see Duncan and Keller 2011). In part one of this paper, we therefore try to create a comprehensive overview and integration of the findings so far, by reviewing 53 published cG × E studies including interactions between the six most studied candidate genes (i.e., the monoamine oxidase A (*MAOA*), the dopamine receptors D4 (*DRD4*) and D2 (*DRD2*), the dopamine transporter 1 (*DAT1*), the 5′ serotonin transporter linked polymorphic region (*5-HTTLPR*), and the catechol-*O*-methyltransferase (*COMT*)) and postnatal family adversity in externalizing behaviors, such as aggression, and conduct disorder.

Another issue concerning cG × E is that there is a lack of insight into biopsychosocial mechanisms that underlie such interactions (see also Battaglia 2012; Dodge 2009; Salvatore and Dick 2015). At present, looking at cG × E findings is like looking at a “black box,” in that we are only aware of what goes in and what comes out. However, insights into how these G × E interactions work (i.e., “how genes get outside the skin,” Reiss and Leve 2007) is of great empirical and clinical importance. From an empirical perspective, it might help us form specific theory-based hypotheses that can specify our research strategies. From a clinical perspective, information on working mechanisms will increase our insight into which proximal variables (i.e., neurobiological and psychological characteristics, rather than genotypes) moderate the effects of family adversity on specific externalizing behaviors. Also, it can increase our knowledge of differential (biological) pathways leading to externalizing problems. This knowledge could in turn be used to tailor interventions by indicating the needed clinical focus, increasing their effectiveness (Matthys et al. 2012). In part two of this paper, we therefore put forward three possible, and complementary, theoretical mechanisms underlying G × E. These proposed mechanisms are based on extant literature about genetic, neurobiological, psychological, and environmental factors within externalizing behaviors.

## Genes, Postnatal Family Adversity, and Externalizing Behaviors: A Systematic Review of G × E Findings

### Methods

This systematic review considers candidate genes that are studied most extensively in the context of externalizing behaviors, namely polymorphisms regulating the activity of the neurotransmitters dopamine and serotonin, which are associated with various aspects of human behavior: the *MAOA*, *DRD4*, *DRD2*, *DAT1*, *5*-*HTTLPR*, and *COMT* val/met. We conducted a literature search for studies on interactions between these polymorphisms and indices of postnatal family adversity in predicting externalizing behaviors (i.e., aggression, behavioral problems, antisocial behavior, Oppositional Defiant Disorder (ODD), Conduct Disorder (CD), delinquency, psychopathy). Our review focus is on externalizing behavior, because these behaviors are relatively common in childhood, and a childhood onset of such problem behavior is known to be a strong predictor of psychopathological outcomes later in life (e.g., Jokela et al. 2009; Von Stumm et al. 2011). However, externalizing behavior is a very heterogeneous behavioral cluster, which has different etiologies in different children and across symptoms (e.g., Frick 2012). Specifically, Attention Deficit Hyperactivity Disorder (ADHD) might be a distinct disorder in symptomatology (i.e., attention deficits) and etiology (e.g., stronger heritability than other externalizing disorders, Burt 2009). Therefore, to narrow the scope of our review, we did not include studies that focused specifically and solely on ADHD as an outcome variable, but did include studies that included ADHD as one of multiple (comorbid) outcome measures. Family adversity (i.e., family and parental characteristics that are associated with increased risks of child maladjustment) is one of the most well-studied and documented contributors to child externalizing behaviors, as well as an important target for interventions aimed at reducing externalizing behaviors (for an overview see Tolan et al. 2013).

We searched digital databases (i.e., PsycINFO, PubMed, Google Scholar) for peer-reviewed papers between January 2002 and May 2015 using the terms: adverse family environment, SES, parent* (the asterisk indicates that the search contained that word base), maltreatment, and psychosocial (environmental factors); G × E, gene–environment, *DRD4, DRD2, MAOA, 5*-HTT*, DAT1, and *COMT* (genetic factors); and all combinations of these factor terms. Also, we searched reference lists of published studies, meta-analyses, and review articles, and contacted authors for possible additional studies. The last search took place on May 1, 2015.

After our original search, 102 studies were selected, of which 49 were excluded because they did not report on externalizing behaviors as defined above as an outcome (e.g., but on ADHD or on externalizing behavior-related constructs such as behavioral disinhibition); did not address *postnatal* family adversity (e.g., but prenatal adversity such as maternal smoking, or risk factors outside the family such as neighborhood); reported exclusively on beneficial family environments, or enrichment of this environment (e.g., maternal warmth or intervention studies: for an overview of RCT’s testing cG × E see Van IJzendoorn and Bakermans-Kranenburg 2015); or reported on interactions that were based on cumulative or polygenic effects only. We did include studies that addressed multiple genes, and findings for all genes were reviewed separately. We also included studies that addressed genetic differential susceptibility rather than genetic risk (i.e., “for better and for worse” interactions; Belsky 2005). It has been hypothesized that the same genetic markers associated with children being relatively vulnerable, and in consequence do worse under environmental adversity (e.g., develop externalizing behaviors), might also be associated with them also being relatively susceptible, and in consequence do better under environmental enrichment (e.g., develop prosocial behavior), compared to children without this marker (Belsky et al. 2007). Moreover, under some supportive environmental circumstances these same markers might even point to a genetic advantageous for children’s development (i.e., vantage sensitivity, Pluess and Belsky 2013). However, because most studies test the cumulative aversive effects of genotype and environment (i.e., dual risk), rather than differential susceptibility or vantage sensitivity, we solely reviewed findings involving environmental adversity (i.e., the “for worse” part). For example, when a study assessed both high (i.e., beneficial) and low (i.e., aversive) responsive maternal caregiving (Nikitopoulos et al. 2014), we only reviewed G × E involving low responsive maternal caregiving. After inclusion, we contacted corresponding authors of the studies in order for them to check the included information, and ask them for possible other studies to include. A limitation of our overview is that we were unable to fully control for a possible “file drawer effect.” Unpublished studies may on average report different results from published studies.

### Results

Our review includes 53 studies. We will discuss the results of the review by polymorphism, starting with a short introduction of the polymorphism in question, stating the number of included studies on this polymorphism and using a “vote counting” procedure for describing the findings (i.e., clustering results in the same direction). Integration of the findings will follow after each results paragraph and in the discussion of part 1. A list of included studies and how they were coded is provided in Table 1.

**Table 1:** Overview of included cG × E studies

Authors	G	E	Outcome	G × E (+/−)	*N*	Age	Sex	Risk allele
Fergusson et al. (2012)	*MAOA*	Adverse childhood circumstances (e.g., childhood maltreatment, family material deprivation; child-report)	Antisocial behavior (self-report) and criminal offending (official records)	+	399	Followed from 15 to 30	M	Low-activity (2.5 and 3 repeats)
Fergusson et al. (2011)	*MAOA*	Childhood abuse or either regular or harsh/severe levels of physical punishment (child-report); interparental violence (Conflict Tactics Scale; child-report)	Property offending (self-report); violent offending (self-report); convictions for property/violent offending (official records); conduct problems (self-report); hostility (SCL-90; self-report)	+	398	Followed from 15 to 30	M	Low-activity (2.5 and 3 repeats)
Weder et al. (2009)	*MAOA*	Trauma (multi-informant Total Trauma Exposure Score)	Aggressive behavior (TRF)	+	114	5–15; *M* = 9.7	M/F	Low-activity (2, 3, and 5 repeats)
Foley et al. (2004)	*MAOA*	Adverse childhood environment (parental neglect; parent-report and exposure to interparental violence and inconsistent parental discipline; child-report)	Conduct disorder (multi-informant child and adolescent psychiatric assessment)	+	514	8–17; *M* = 12.23	M	Low-activity (2, 3, and 5 repeats)
Prom-Wormley et al. (2009)	*MAOA*	Childhood adversity (parental neglect; parent-report and exposure to interparental violence or inconsistent parental discipline; child-report)	Conduct disorder (multi-informant Child and adolescent psychiatric assessment)	−^a^	721	8–17; *M* = 11.24	F	N.A.
Widom and Brzustowicz (2006)	*MAOA*	Neglect and abuse (official records)	Violent and antisocial behavior (number of arrests, self-reports, and psychiatric interview)	+ (White subsample)/−(other subsamples)	631	Adults	M/F	Low-activity (males with one or females with two 3-repeat)
Haberstick et al. (2005)	*MAOA*	Maltreatment (child-report)	Conduct problems (measure of DSM-IV criteria, self-report, and violent convictions)	−	774	Followed from 16 to 22	M	N.A.
Caspi et al. (2002)	*MAOA*	Maltreatment (behavioral observations of e.g., harshness, parental reports, and retrospective reports)	Composite index of antisocial behavior: (1) conduct disorder; DSM-IV, (2) convictions for violent crimes; official records, (3) disposition toward violence; self-report; (4) sAntisocial personality disorder; friend/family report	+	442	Followed from 3 to 26	M	Low-activity (2 or 3 repeats)
Huizinga et al. (2006)	*MAOA*	Maltreatment (child-report)	Life course problem behavior and violence and composite index of antisocial and violent behavior: (1) conduct disorder; DSM-IV, (2) arrests; official records and self-report, (3) disposition to violence; self-report (4) sAntisocial personality disorder; self-report and parent/spouse report	−	277	Followed from 11 to 28^b^	M	N.A.
Prichard et al. (2008)	*MAOA*	Childhood adversity (child-report)	Antisocial behavior (using indicator variables; self-report)	−	1002	Adults	M	N.A.
Derringer et al. (2010)	*MAOA*	Harsh discipline and sexual abuse (child-report)	Antisocial behavior and conduct disorder (lifetime prevalence and symptom count; DSM-IV)	+^c^	841	Followed from 11 (cohort 1) or 17 (cohort 2)	M/F	Low-activity (2, 3 or 5 repeats)
Åslund et al. (2011)	*MAOA*	Maltreatment (summation index; child-report)	Delinquency (self-report)	+	1825	17 or 18	M/F	Low-activity (boys: 2 or 3 repeats)/high-activity (girls: 3.5, 4, or 5 repeats and heterozygous: low/high)
Van der Vegt et al. (2009)	*MAOA*	Early maltreatment (before adoption reported by adoptive parents)	Externalizing behavior (CBCL)	−	239	10–15	M	N.A.
Ducci et al. (2007)	*MAOA*	Childhood sexual abuse (psychiatric interview and medical records child)	Antisocial personality disorder (DSM-IV) or sAntisocial personality disorder (schedule for affective disorders and Schizophrenia-lifetime version)	+	291	Adults	F	Low-activity (homozygous)
Young et al. (2006)	*MAOA*	Maltreatment (Colorado Adolescent Rearing Inventory; child-report)	Conduct disorder (DISC; self-report)	−	247	12–18	M	N.A.
Kim-Cohen et al. (2006)	*MAOA*	Maltreatment (child-report)	Behavior problems (CBCL, TRF, and child-report)	+^d^	975	7	M	Low-activity (2, 3, and 5 repeats)
Frazzetto et al. (2007)	*MAOA*	Early traumatic life events (related to family environment; child-report)	Physical aggression (self-report)	+(Males)/−(females)	90	Adults	M/F	Low-activity (2, 3 and 5 repeat)
Sjöberg et al. (2007)	*MAOA*	Psychosocial factors: type of housing and experiences of childhood sexual abuse (child-report)	Criminal activity (self-report)	+	119	16 or 19	F	High-activity (4 repeat)
Beach et al. (2010)	*MAOA*	Maltreatment (child-report)	Antisocial personality disorder (maximum lifetime symptom levels of antisocial personality disorder: DSM–III–R; using SSAGA–II)	+	538	Adults	M/F	Low-activity (3 repeat)
Nilsson et al. (2006)	*MAOA*	Psychosocial factors: type of housing and experiences of childhood sexual abuse (child-report)	Criminal activity (self-report)	+	81	16 or 19	M	Low-activity (3 repeat)
Vanyukov et al. (2007)	*MAOA*	Parenting (child assessment of parental involvement and behavior)	Psychiatric diagnosis: kiddie-schedule for affective disorders and Schizophrenia (mother and child-report)	+^e^	144	Followed from 10 to 19	M	High-activity (3.5 and 4 repeats)
Edwards et al. (2010)	*MAOA*	Physical discipline (Conflict Tactics Scale; mother-report)	Externalizing behavior (CBCL, YSR/YASR, and TRF)	+^f^	186	Followed from 6 to 22	M	Low-activity (2, 3, or 5 repeats)
Hart and Marmorstein (2009)	*MAOA*	Neighborhood poverty (i.e., the percentage of individuals in the census tract living in households with incomes below the federal poverty level)	Aggression (self-report)	−	579	11–21	M	N.A.
McGrath et al. (2012)	*MAOA*	Childhood physical maltreatment (Childhood Trauma Questionnaire; child-report)	Problem behavior factors: (1) conduct problems (self-report); (2) impulsive sensation seeking (self-report); (3) interpersonal aggression (self-report)	+^g^	192	Adults	F	High-activity (3.5 and 4 repeats)
Reti et al. (2011)	*MAOA*	Childhood physical abuse (child-report)	Antisocial personality (international personality disorder examination)	+	742	Adults	M/F	Low-activity (2, 5, or 3 repeat)
Kinnally et al. (2009)	*MAOA*	Parental care (parental bonding instrument) and early life stressors (clinical assessment Interview, child-report)	Impulsivity and aggression (Brown-Goodwin Aggression Inventory; self-report, Barratt Impulsivity Scale; self-report, and BDHI; self-report)	+^h^	159	Adults	F	High-activity (3.5 or 4 repeat)
Choe et al. (2014)	*MAOA*	Maternal punitiveness (early parenting coding system; observation). Parental punitive discipline (Child Misbehavior Questionnaire; parent-report).	Violent attitudes (Attitudes Towards Violence Scale; self-report), antisocial behavior (Self-Report of Delinquency Questionnaire; peer- and self-reports)	+	189	Followed from 1.5 to 20	M	Low-activity (3 repeat)
Bakermans-Kranenburg and Van IJzendoorn (2006)	*DRD4*	Maternal insensitivity (Ainsworth’s rating scale)	Externalizing behavior (CBCL)	+^i^	47	10 months (maternal sensitivity); 29 months (CBCL)	M/F	7-repeat
Propper et al. (2007)	*DRD4*	Parenting quality (observation of sensitivity/responsiveness, detachment/disengagement)	Externalizing problems (CBCL)	+(African American subsample/−(other subsamples)	169	Followed from 3 to 30 months	M/F	Short (2–6 repeats)
Windhorst et al. (2015)	*DRD4*	Maternal sensitivity (Ainsworth’s rating scales at 14 months/Erickson scales at 36 and 48 months; observation)	Externalizing behavior (CBCL)	+^j^	548	Followed 14 months to 5 years	M/F	7-repeat
Marsman et al. (2013)	*DRD4*	Parental rearing practices (EMBU-C; parent-report)	Externalizing problems (CBCL and YSR)	+	2230	Followed from 10 to 16 (T1 10–12; *M* = 11.09)	M/F	4-repeat
Martel et al. (2011)	*DRD4*	Parenting (APQ; parent-report) and child perception of interparental conflict (child-report)	ADHD or ODD; sADHD (parent and teacher report on the ADHD Rating Scale); ODD symptoms (teacher-report)	+^k^	548	6–18; *M* = 11.67	M/F	Long (homozygous)
Nikitopoulos et al. (2014)	*DRD4*	Early maternal care (observed and coded using category system for microanalysis of early mother–child interaction)	Externalizing symptoms (K-SADS, parent and adolescent interview); Psychopathic behaviors (the psychopathy screening device, parent report)	+	296	Followed to 15	M/F	7-repeat
Schlomer et al. (2015)	*DRD4*	Maternal hostility (child-report)	Aggressive behavior problems (CBCL)	+	580	Followed from 11 to 16	M/F	7-repeat
DeLisi et al. (2009)	*DRD2*	Criminal father (respondent’s biological father had ever been incarcerated yes/no)	Delinquency: police contacts and self-report on serious and violent delinquency	+^l^	232	12–17 (T1); 13.5–18.5 (T2)	F	A1
Thompson et al. (2012)	*COMT*	Maternal stress (The Perceived Stress Scale; mother-report)	Externalizing behavior (SDQ; parent-report)	+	546	Followed from 1–11	M/F	Met (homozygous)
Nobile et al. (2010)	*COMT*	SES (i.e., parental employment according to the Hollingshead scale for parental occupation)	ADHD, ODD and CD problems (CBCL)	+^m^	172	11–14	M/F	Val (homozygous)
Wagner et al. (2010)	*COMT*	Physical maltreatment, rape and childhood sexual abuse (self-report)	Aggression (BDHI; self-report)	+	112	Adults	F	Val (homozygous)
Li and Lee (2010)	*5*-*HTTLPR*	Maltreatment (child-report)	Antisocial behavior (self-report)	+	2488	Followed from 12 to 27 (T1 12–20; *M* = 15.6)	M/F	S
Sadeh et al. (2010)	*5*-*HTTLPR*	Study 1: disadvantaged environments (composite SES index: annual family income and parental occupation scores)	Psychopathic tendencies (APSD; self-report)	+^n^	118	9–17*; M* = 14.3	M/F	L (homozygous)
		Study 2: composite SES index: annual family income (continuous), mother’s educational attainment, and father’s educational attainment.	Callous unemotional traits (inventory of callous- unemotional traits; self-report)	+	178	8–15*; M* = 10.8	M/F	L (homozygous)
Davies and Cicchetti (2013)	*5*-*HTTLPR*	Maternal unresponsiveness (adult–adolescent parenting inventory; maternal report and observational ratings of mother–child interactions: Iowa Family Interaction Rating Scales)	Externalizing behavior (California Child Q-Set and Caregiver–Teacher Report Form; experimenter-report)	+(African American subsample)/−(other subsamples)	201	2	M/F	L (homozygous)
Agnafors et al. (2013)	*5*-*HTTLPR*	Maternal stress and depression (The Edinburgh Postnatal Depression Scale (EPDS); Life Stress Score (LSS))	Externalizing behavior (CBCL)	−	889	Followed to 12	M/F	N.A.
Reif et al. (2007)	*MAOA, DAT1, 5*-*HTTLPR*	Adverse childhood environment: social status, family structure, emotional family climate, social integration, and school education (rated by independent investigator)	Habitual aggressive and violent behavior (expert consensus)	+(*5*-*HTTLPR*)/−(*MAOA, DAT1*)	184	Adults	M	S
Cicchetti et al. (2012)	*MAOA* (boys only), *5*-*HTTLPR*	Maltreatment (MCS; all available information coded by research assistants)	Antisocial behavior (TRF, peer rating, and The Pittsburg Youth Survey; self-report)	+^o^	627	10–12; *M* = 11.27	M/F	Low-activity (*MAOA*; 2, 3 and 5 repeats)/S homozygotes (*5*-*HTTLPR*)
Simons et al. (2011)	*DRD4, 5*-*HTTLPR*	Social environment (Harsh parenting; self-report)	Aggression (DISC; self-report at wave 1 and Elliott’s Instrument self-report at wave 5).	−^p^	505	Followed from 12 to 21	M/F	N.A.
Sonuga-Barke et al. (2009)	*DAT1, DRD4, 5*-*HTTLPR*	Parental expressed criticism (coded by researchers using codings derived from the Camberwell Family Interview)	Conduct problems (SDQ; parent and teacher-report)	+(*DAT1, 5*-*HTTLPR*)/−(*DRD4*)	673	5–17	M	9-repeat (*DAT1*)/S (*5*-*HTTLPR*)
Nederhof et al. (2012)	*COMT, DRD4, DRD2*	Parental separation/divorce before the age of 16	Externalizing behavior (YSR)	+(*COMT, DRD4*)/−(*DRD2*)	1134	16	M/F	Met (*COMT*)/7-repeat (*DRD4*)^q^
Nobile et al. (2007)	*DRD4, 5*-*HTTLPR*	Social economic status (Hollingshead scale for parental occupation)	Rule-breaking and aggressive Behavior (CBCL)	+	589	10–14; *M* = 12.0 (boys)/*M* = 12.13 (girls)	M/F	L (homozygous) (*5*-*HTTLPR*)/L (*DRD4*; 6-8 repeats)
Beaver et al. (2012)	*DRD4, DRD2*	Neighborhood disadvantage^r^ ((a) the proportion of households that were single-parent headed (b) the proportion of households with an income <US$15,000 (c) the proportion of households receiving public assistance (d) the proportion Black and (e) the unemployment rate)	Violent delinquency (self-report)	+	1026	12–21; *M* = 16.1	M/F	7-repeat (*DRD4*)/A1 (*DRD2*)
Sadeh et al. (2013)	*MAOA, 5*-*HTTLPR*	Childhood abuse (The Childhood Trauma Questionnaire)	Psychopathic traits (The Psychopathy Checklist: Screening Version; self-report)	−	237	Adults	M	N.A.
Lavigne et al. (2013)	*5*-*HTTLPR, DRD4, MAOA*	Risk factors: Social economic status (Hollingshead Four-Factor Index of social status), parental stress (Perceived Stress Scale; Parenting Stress Index Short Form, parent report), family conflict, caretaker depression, Parental support and hostility, Support/scaffolding (observed)	Childhood externalizing psychopathology (Diagnostic Interview Schedule for Children–Parent Scale—Young Child (DISC-YC); child symptom inventory (CSI); Eyberg Child Behavior Inventory (ECBI); parent report)	+(*5*-*HTTLPR*)^s^/−(*DRD4, MAOA*)	175	4–5; *M* = 4.40	M/F	L (homozygous)
Richards et al. (2015)	*5*-*HTTLPR, DRD4, DAT1*	Maternal expressed criticism (rated by interviewer)	Antisocial behavior (SDQ, parent- and self-report)	−	366	8–28; *M* = 17.11	M/F	N.A.
Boardman et al. (2014)	*DAT1, DRD2*	Family closeness (reversed coded, self-report)	Serious and violent delinquency (self-report)	+	724	12–17 at timepoint 1 (followed 27 years)	M/F	10R (*DAT1*)/A1 (*DRD2*)

*Note* Some studies included multiple environmental and/or outcome measures. Only details and findings on postnatal family adversity and externalizing behavior were shown in this table

^a^Initial interaction-effect with the high-activity allele (3.5 and 4 repeat) was no longer significant after adjusting for sample sizes at each level of childhood adversity

^b^Multiple birth cohort

^c^Significant G × E only with sexual abuse and *MAOA* in predicting antisocial behavior and conduct disorder symptoms

^d^Significant G × E only with physical abuse

^e^The strength and direction of relationships depended on the parental sex. The G × E was not detected using stratified analyses, conducted in the genotypic classes separately

^f^Significant G × E only on delinquency scale

^g^Significant G × E only with *MAOA* and physical maltreatment on conduct problems

^h^E × E × G: better perceived parental care offset the effects of life stressors in carriers of the low-activity allele, but not in carriers of the high-activity allele

^i^Replicated in twin siblings

^j^Significant interaction with sensitivity at age 14 months only: differential susceptibility for externalizing behavior at 18 months, but contrastive effects for 7-repeat carriers and non-carriers at 36 months

^k^Significant G × E only on ADHD diagnosis

^l^Null findings (not shown in paper) were found for African American males, Caucasian males and females

^m^Significant G × E only on ADHD problems

^n^Significant G × E only on Callous Unemotional and Narcissistic scales

^o^ *5*-*HTTLPR* × maltreatment on all outcomes; *MAOA* × maltreatment on self-report conduct symptoms in boys only

^p^Only cumulative genetic effects were found

^q^Opposite effects for boys and girls (protective model boys, dual risk model girls)

^r^Social and economic adversity

^s^ *5*-*HTTLPR* × family stress only

#### MAOA

The *MAOA* gene codes for the monoamine oxidase A enzyme, which is involved in the degradation of dietary amines and neurotransmitters, such as serotonin and dopamine. The gene is located on the X-chromosome, this means that women have two alleles and men have only one. The *MAOA* polymorphism is a Variable Number Tandem Repeat (i.e., VNTR polymorphism) in the promoter region of the gene starting 43,515,409 basepairs from the end of the chromosome (pter), comprising a 30-basepair repeat sequence present in 2, 2.5, 3, 3.5, 4, 5, or 6 copies (Sabol et al. 1998). The 2 and 3-repeats are indicated as “low-activity variant” and the 3.5 and 4-repeat sequences as “high-activity variant.” Although distribution varies among different populations, the 3 and 4-repeats are usually the most prevalent. To date, there is no consensus on the activity level of the less-prevalent 2.5 and 5-repeat (see Deckert et al. 1999), and the 6-repeat has not been functionally characterized. The high-activity alleles code for higher transcription of monoamine oxidase A, resulting in an increased degradation—and thus decreased concentrations—of dopamine and serotonin in the brain (Denney et al. 1999; Sabol et al. 1998). Dopamine is involved in, among others, motivation, motor control, and cognition (Missale et al. 1998) and serotonin in memory, learning, and mood (Pezawas et al. 2005). A decreased concentration of dopamine and serotonin is linked to impulsivity, antisocial behavior, and alcoholism (e.g., Eme 2013; Schmidt et al. 2000). Furthermore, the *MAOA* polymorphism has also been directly related to antisocial behavior (for a meta-analysis, see Ficks and Waldman 2014). See for an overview of dopamine-related cG × E Bakermans-Kranenburg and Van IJzendoorn (2011).

We found 31 studies including the *MAOA* polymorphism, family adversity, and externalizing behaviors (Table 1). The original Caspi et al. (2002) finding that the effect of family adversity on externalizing behaviors is larger among low-activity allele carriers has been replicated 16 times. We found four studies that reported this effect to be larger among high-activity allele carriers, and we found ten null findings (i.e., no interaction effect). It is important to note, however, that studies replicating the original interaction differed in how they operationalized the low-activity allele—sometimes as 3; as 2 and 3; as 2.5 and 3; or as 2, 3, and 5-repeat sequences. A recent meta-analysis of Byrd and Manuck (2014) shows a moderately consistent interaction between the low-activity allele and maltreatment in predicting conduct problems in males. For other environmental adversities, however, the interaction was found to be less consistent. This might indicate that specific polymorphisms interact with specific environmental factors, in predicting specific externalizing behavior. For example, the *MAOA* might interact with harsh parenting and maltreatment (e.g., Weder et al. 2009), through a specific mechanism of vulnerability predicting antisocial and aggressive behavior, but might not necessarily interact with other environmental factors such as poverty (e.g., Hart and Marmorstein 2009), and might not necessarily predict other forms of externalizing behavior (e.g., psychopathic traits, Sadeh et al. 2013).

Opposed to male populations, in female populations there was a significant interaction found between environmental adversity and the *high*-*activity* allele in predicting externalizing behaviors. We know little about how these sex differences might be explained, although the literature does suggests different possibilities. First, the differences might be partly due to the fact that the *MAOA* is an X-linked polymorphism. The role of *MAOA* genotype on *MAOA* expression might be more unpredictable for women than for men (e.g., Carrel and Willard 2005; Pinsonneault et al. 2006). Second, *MAOA* expression might be affected by sex hormones such as testosterone (Ou et al. 2006; Sjöberg et al. 2007). High testosterone levels may lead to lower transcription of *MAOA* and lower *MAOA* levels. Because of higher testosterone levels, men might show lower levels of *MAOA* in general, which in turn might have larger effects on dopamine availability and in turn behavior, compared to women. This explanation might be specifically interesting for studies addressing adolescence, when testosterone levels are particularly high. Third, the effect or prevalence of the environmental risk factor, and/or the mechanism underlying the interaction between environmental risk and *MAOA* genotype, might be different for boys than for girls (Beach et al. 2010). For example, within the broad measure of childhood maltreatment, neglect might be a particularly important risk factor for externalizing behavior in boys, while sexual abuse is more important for girls.

#### DRD4

The *DRD4* gene codes for the D4 subtype of dopamine receptors (i.e., dopamine binding sites) in the brain. The *DRD4* polymorphism is a VNTR in exon 3 of the gene starting 637,293 basepairs from pter, comprising a 48 nucleotide repeat sequence ranging from 2 to 11 copies (Van Tol et al. 1992). The common 2–5 repeats are indicated as “short” and 6–10 repeats as “long” variants of the polymorphism. The long-allele is associated with significantly reduced amounts of D4 receptors in the brain (Asghari et al. 1995). Specifically, the relatively common 7-repeat allele is related to a blunted dopamine response (Schoots and Van Tol 2003), which has been related to a reduced reward processing (for a review see Comings and Blum 2000). Lower amounts of dopamine receptors have been consistently found in people suffering from substance abuse (Li et al. 1997; Volkow et al. 1997). Polymorphisms coding for lower amounts of dopamine receptors seem to moderate the effects of environmental adversities on the development of different forms of psychopathology (for a meta-analyses see Bakermans-Kranenburg and Van IJzendoorn 2011).

We found 14 studies including the *DRD4* polymorphism, family adversity, and externalizing behaviors (Table 1). Nine studies found the effect of family adversity on externalizing behaviors to be larger among carriers of the long-allele, of which seven studies found this specifically for the 7-repeat allele. Two studies found this effect to be larger among carriers of the short-allele, of which one study found this specifically among 4-repeat allele carriers and one study among carriers of the short-allele operationalized as 2–6-repeats. Three null findings have been published. Some of these inconsistencies between findings might be due to differences in sample composition between studies, specifically differences in sample age. The mean sample age of studies on the *DRD4* varies between 10 months at the first time point of a longitudinal study and around 16.5 years at the last measurement point of a longitudinal study. It might be that certain cG × E are age specific: There might be critical (i.e., restricted developmental periods in which influences of a particular G × E occurs) and sensitive (i.e., developmental periods in which influences of a particular G × E are more likely to occur) periods (see for a critical discussion and examples, Reiss et al. 2013). For example, Windhorst et al. (2015) found the *DRD4* genotype to moderate the relation between maternal insensitivity at 14 months and externalizing behavior at 18 months, but not at 48 months (i.e., at the ages of 48 months and up maternal insensitivity predicted child externalizing behavior, independent of *DRD4* genotype). Moreover, for different age groups, different assessment tools exist. Therefore, such age differences might bring about differences in the measurements used to assess the environment and/or behavioral outcomes.

Sex differences have also been reported. Opposite effects of the *DRD4* polymorphism have been found for boys and girls (i.e., protective model for boys and dual risk model for girls; Nederhof et al. 2012). It might very well be that the mechanisms underlying such interactions are different for boys and girls. Another explanation might be that some cG × E are explained by confounding effects of covariates such as gender, ethnicity, or social economic status (see, e.g., Keller 2014). For example, Dmitrieva et al. (2011) found that the gender-specific direct effects of the *DRD4* on externalizing behavior were explained by differences in exposure to family adversity (i.e., poor parental monitoring and exposure to violence). Some cG × E regarding the *DRD4* might therefore be explained by a gender-by-adversity or a gender-by-*DRD4* interaction. In the case of monitoring, the literature suggests that parental monitoring is a stronger predictor of externalizing behavior in boys, than in girls (Jacobson and Crockett 2000), and that boys receive less parental monitoring than girls (e.g., Webb et al. 2002), specifically when they carry the *DRD4* 7-repeat allele (Dmitrieva et al. 2011).

#### DRD2

The *DRD2* gene codes for the D2 subtype of the dopamine receptors. The Taq1A *DRD2* polymorphism is a single nucleotide polymorphism (SNP) (rs 1800497) of the gene, resulting in a cytosine (C) to thymine (T) substitution. The less frequent A1-allele (T) is associated with significantly reduced amount of D2 receptors in the brain compared to the A2-allele (C) (Noble et al. 1991), which might result in a blunting of dopamine signals. The A1-allele is associated with impulsivity (Eisenberg et al. 2007). However, more recently, this polymorphism has been more precisely located within the coding region of a neighboring gene (10 kb downstream the *DRD2* gene), named *ANKK1. ANKK1* activity may provide an alternative explanation for previously described associations between the *DRD2* and neuropsychiatric disorders (Neville et al. 2004).

We found four studies including the *DRD2* polymorphism, family adversity, and externalizing behaviors (Table 1). One study reported a null finding. Three studies found a significant interaction effect, in which the effects of family adversity were larger among carriers of the A1-allele. The three significant findings all related to interactions between the *DRD2* and family dysfunctioning on adolescent delinquency (Beaver et al. 2012; Boardman et al. 2014; DeLisi et al. 2009). One study that did not find a significant interaction focused on the interaction between the *DRD2* and early parental separation on externalizing behavior (i.e., YSR, Nederhof et al. 2012). The different findings between these studies might therefore be explained by differences in the conceptualization of family adversity and/or externalizing behavior outcome. Children’s *DRD2* genotype might not affect the relation between parental separation—which might not necessarily correlate with the experience of family dysfunctioning—and externalizing behavior, whereas it does affect the relation between family adversity measures, such as the experience of having an incarcerated father or a lack of family closeness, and delinquency. Alternatively, the *DRD2* might interact with family adversity in predicting adolescent delinquency, but not in predicting other or broader forms of externalizing behavior.

#### DAT1

The *DAT1* gene (SLC6A3) regulates the uptake of dopamine by influencing the quantity of dopamine available in the synapses in the brain (i.e., striatum, prefrontal cortex, and hypothalamus). The *DAT1* polymorphism is a VNTR on the 3′-untranslated region of the gene starting 1,392,905 basepairs from pter, comprising a 480 basepair repeat sequence varying between 3 and 11 copies. The 9 and 10-repeat are the most common variants (VanNess et al. 2005). Lower expression of the *DAT1* is related to lower dopamine availability in the synapses of the brain. There are conflicting findings regarding the expression levels of the 9-repeat and 10-repeat alleles (e.g., Heinz et al. 2000; Van Dyck et al. 2005). The 9-repeat is (population specifically) associated with addiction (Bhaskar et al. 2012), whereas the 10-repeat allele is associated with impulsivity (for a meta-analysis, see Yang et al. 2007).

We found four studies including the *DAT1* polymorphism, family adversity, and externalizing behaviors: Two studies reported a null finding and two studies found a significant interaction (Table 1). These latter two studies, however, both reported larger effects of family adversity among carriers of different *DAT1* variants (i.e., the 10-repeat allele or the 9-repeat allele). Again, an explanation might be found in the large differences in family adversity measures, which ranges from parental expressed criticism, and global institutional deprivation, to family closeness. Studies might be incomparable because they simply test relations between different constructs. It is questionable if similar outcomes are to be expected between studies testing interactions with such different environmental adversity and outcome measures.

#### COMT

The *COMT* gene codes for the *catechol*-*O*-*methyltransferase* enzyme, which breaks down catecholamines including dopamine, thus clearing them from the synapse. The *COMT* polymorphism is a SNP (rs 4680) resulting in a valine (i.e., Val) to methionine (i.e., Met) mutation. The Val-allele is related to higher activity than the Met-allele—with differences up to 400 %—leading to lower synaptic dopamine levels (e.g., Chen et al. 2004; Lotta et al. 1995). This polymorphism is related to individual differences in emotional processing (stronger activation of the prefrontal cortex in Met-allele carriers) and cognitive processing (reduced prefrontal cortex efficiency for Val-carriers) (for a meta-analysis, see Mier et al. 2009). A meta-analysis showed no direct associations between the *COMT* polymorphism and externalizing psychopathology (Munafò et al. 2005), but indications were found that heterozygosity serves as a protective factor for psychopathology (Costas et al. 2011).

We found four studies including the *COMT* polymorphism, family adversity, and externalizing behaviors (Table 1). Two studies found the effect of family adversity to be larger among carriers of the Met-allele and two studies among carriers of the Val-allele. For example, Wagner et al. (2010) showed that women who carried the Val-allele—and had been maltreated during their childhood—manifested more hostile antisocial behavior compared to non-carriers. In contrast, Thompson et al. (2012) found the effects of maternal stress on externalizing behavior to be larger for children homozygous for the Met-allele than for children with the Val-allele. In the case of the *COMT* polymorphism, this seemingly contradiction might be explained by a cognitive/emotional trade-off (i.e., the warrior–worrier hypothesis, Goldman et al. 2005), in which the Val-allele is associated with an advantage in emotional processing and the Met-allele in cognitive processing (see Mier et al. 2009). The Met-allele (i.e., the worrier) has been associated with an advantage for prefrontal cortex—and related cognitive—functioning. However, at the same time this allele might form a genetic predisposition for heightened emotional arousal and affective responses, and lower emotional control, which might contribute to emotional dysregulation, an irritable mood and externalizing behavior reported in Met/Met individuals (e.g., negative mood, irritability and affective disorders) (e.g., Drabant et al. 2006; Williams et al. 2010; Thompson et al. 2012). The Val-allele (i.e., the warrior) has been associated with better stress and anxiety resistance, but lower executive functions (Wishart et al. 2011) and cognitive control (Kilford et al. 2015), which might contribute to deficits in response inhibition, and substance dependence in Val-carriers (e.g., Nobile et al. 2010; Gratacòs et al. 2007). The two different alleles might therefore both function as genetic risk and/or advantage under different environmental adversity.

#### 5-HTTLPR

The *5*-*HTT* gene (SLC6A4) codes for serotonin transporters, which are involved in the active clearance and termination of synaptic serotonin. The *5*-*HTT* gene linked polymorphic region, or *5*-*HTTLPR,* is a polymorphism in the promoter region of the gene. The *5*-*HTTLPR* starts 28,521,337 basepairs from pter and consists of a 20–23 basepair repeat sequence. The two most common variants of the polymorphism are typically defined as a “short-allele” (i.e., S-allele, low expressing) comprising 14 copies and a “long-allele” (i.e., L-allele, high expressing) comprising 16 copies. The S-allele has been related to significantly lower 5-HTT mRNA and protein, lower uptake and consequently higher and less stable concentrations of serotonin in the synaptic cleft, compared to the L-allele (e.g., Greenberg et al. 1999; Lesch et al. 1996, but for contrasting findings see Naylor et al. 1998). Serotonin is indicated as an important modulator of neural circuitry that controls a wide range of behavioral and physiological processes including mood (e.g., Pezawas et al. 2005). The *5*-*HTTLPR* polymorphism is intensely studied in association with internalizing problems (e.g., Uher and McGuffin 2007), but has also been related to other forms of child and adolescent psychopathology (see Van IJzendoorn et al. 2012).

We found 12 studies including the *5*-*HTTLPR* polymorphism, family adversity, and externalizing behaviors (Table 1). Four studies found the effect of family adversity to be larger among carriers of the S-allele. In contrast, four studies found this to be larger among carriers of the L-allele. These findings might indicate that both the *5*-*HTTLPR* S-allele and the L-allele might contribute to externalizing behavior after exposure to family adversity, through different pathways. The S-allele might be related to increased neural activity (e.g., Murphy et al. 2013), affective and physiological reactivity (e.g., Gyurak et al. 2013, but for contrasting findings see Weeland et al. 2015), and lower levels of positive affect (e.g., Hankin et al. 2011), after exposure to negative emotions. This heightened emotional reactivity might form a risk for irritability and reactive aggression when exposed to negative emotions (e.g., Cicchetti et al. 2012), whereas the L-allele might be related to emotional hyporeactivity and punishment insensitivity (for a review see Glenn 2011), and might therefore be a risk for proactive and predatory behavior when socialization is mainly based on punishment (e.g., Sadeh et al. 2010).

Four studies reported a null finding. However, this number might be misleading since it has been show that specifically the literature on the *5*-*HTTLPR* in externalizing behavior might suffer from a publication bias in favor of statistically significant findings (Ficks and Waldman 2014). Also, ethnic differences have been found. Specifically, Davies and Cicchetti (2013) found that African American 2-year-olds—but not 2-year-olds from other ethnic subgroups—homozygous for the L-allele were more susceptible to maternal unresponsiveness in developing externalizing behaviors, compared to carriers of the S-allele. The L-allele has been shown to be more common among African Americans, compared to other ethnic groups (Enoch et al. 2006). Such differences in allele frequency might be important for several reasons. First, it might bring about differences in group sizes regarding the relevant genotypes (e.g., a relative larger group of L-carriers in African American samples than equally large samples of other ethnicity), and therefore power, between studies with different sample compositions. Second, due to possible gene–environment or gene–behavior correlations, it might cause differences between study samples in the prevalence of the specific environmental risk factor and/or outcome behavior.

### Discussion

The initial stage of the cG × E research field has yielded an interesting set of results, detecting many interaction effects between genetic polymorphisms and family adversity on externalizing behaviors. Our review of this literature shows a large set of studies that feature mixed results (see Table 1). To illustrate, for the *5*-*HTTLPR* VNTR polymorphism, null findings (4 out of 12) as well as interactions with both the S-allele (4 out of 12) and L-allele (4 out of 12) as “risk allele” have been reported. Furthermore, heterogeneity of results might be underestimated due to a publication bias in this field favoring statistically significant findings over nonsignificant findings (see Duncan and Keller 2011; Ficks and Waldman 2014). Based on this, one might conclude that cG × E has failed to deliver conclusive evidence for specific cG × E. However, our review also makes clear that—even though we specified our search terms by means of a specific environmental factor and outcome—most studies have large methodological differences, which makes it difficult to make cross-study comparisons of findings. Findings on cG × E concerning the *MAOA* polymorphism seem to be an exception, showing a relatively consistent pattern. This might be partly explained by the fact that studies on this polymorphism have been using—more so than studies on other polymorphisms—more similar measures for family adversity (i.e., abuse and maltreatment) and outcome behavior (i.e., antisocial behaviors and conduct disorder).

The methodological differences between studies largely fall under three categories, namely sample size and composition, conceptualization, and power. These differences have been addressed extensively before (see for a critical review Dick et al. 2015; Jaffee et al. 2013) and therefore will only be discussed briefly. First, our table shows that both sample size (i.e., the *N* ranged from 47 to 2488) and composition—in terms of sex, ethnicity/geography, and age—vary strongly between studies. This is important, given that differential genetic effects should be expected by sex (e.g., Nordquist and Oreland 2010) and ethnicity or geography, the latter based on population stratification (e.g., Manica et al. 2005; Enoch et al. 2006). Some of the inconsistencies between findings might be explained by ethnic differences in allele frequency (e.g., Enoch et al. 2006), sex and/or ethnic differences in the prevalence of specific externalizing behaviors and environmental risk factors (e.g., Miner and Clarke-Stewart 2008), or differences in the mechanisms underlying externalizing behavior (see Deater-Deckard and Dodge 1997). Therefore, specific cG × E found in one population might not necessarily be replicated in another. Age also plays an important role, given that across age groups different tools are being used to assess the environment and behavioral outcomes. Moreover, different predictors play a key role across different developmental stages (Moffitt et al. 2006). Thus, again a specific cG × E found within one developmental period might not necessarily be replicated in another. This is especially relevant in light of the fact that previous longitudinal cG × E studies have targeted samples within a broad range of different developmental periods. More specifically, it might be that there are sensitive periods for specific cG × E (Belsky and Pluess 2013; Reiss et al. 2013). An example of such “timing effects” for cG × E involving the *MAOA* can be found in Choe et al. (2014) and involving the *DRD4* in Windhorst et al. (2015). Moreover, it has also been found that the effects of a polymorphism on brain function might be age dependent (e.g., Meyer et al. 2014). Future research should therefore not only focus on if and how specific cG × E occurs, but also on the timing of specific cG × E (i.e., when it occurs).

Second, our review shows that studies on cG × E are characterized by a large diversity in the conceptualization of, and type of measures used to assess, both family adversity and externalizing behaviors (e.g., present vs. retrospective; self-reported vs. observed behavior). Moreover, disparate concepts are sometimes described using the same terminology. To illustrate, antisocial behavior has been assessed with the number of arrests, as well as the amount of DSM-related symptoms. This issue is not specific to literature on cG × E. However, although it is important that original findings are extended by broadening the scope of predictors, outcomes, and populations, these large differences between studies makes it difficult to make cross-study comparisons. This is important since new hypotheses on G × E are often based on previous findings, regardless of these differences. This approach might not be specific enough, leading to hypotheses based on inadequate literature or theory. Also, this diversity raises a fundamental question within the field of G × E: Do we expect a polymorphism to be related to individual differences in susceptibility to the environment in general, or to individual differences in susceptibility to specific environmental factors through specific risk mechanisms? And also, do G × E underlie psychopathology in general or do they underlie specific psychopathological outcomes? The inconsistent findings between different studies seem to suggest specific rather than general interactions between genes and environment. Moreover, which allele of a specific polymorphism should be considered the “risk” allele for externalizing behavior might be dependent on the specific study population, adversity measure, and outcome measures used. We gave specific examples of how both alleles of the *COMT* and *5*-*HTTLPR* polymorphisms, through different mechanisms, might form a risk factor for different externalizing behaviors. Moreover, some alleles might even form a “risk” factor for specific externalizing behavior when exposed to certain environmental adversity, while at the same time being “advantageous” when exposed to other environmental factors (e.g., the emotional/cognitive trade-off in case of the *COMT*). Third, the described differences in sample size and conceptualization bring about differences in statistical power (see Caspi et al. 2010; Duncan and Keller 2011), affecting the a priori likelihood that studies will come up with significant and replicable cG × E findings (see also Simmons et al. 2011).

Given these large methodological differences between the studies, different findings are simply to be expected and do not necessarily illustrate that findings are contradictory. One way to help build a more consistent literature base, to establish better comparable findings, and to consequently draw more solid conclusions about the role of cG × E in externalizing behaviors, might be through specifying a priori hypotheses: How do specific family adversity factors interact with specific genetic polymorphisms, in predicting specific behavior in specific samples, and in turn by stating our research strategies accordingly. This approach will also reduce the chance of spurious findings due to chance capitalization (Type I errors) and will allow scholars to conduct a priori power analyses, reducing chances of false negatives (Type II errors). To be able to state adequate hypotheses however, we need to move away from the exploratory phase and form a more biologically informed understanding of these interactions. Insight into mechanisms underlying G × E might help us form theory-based hypotheses, for example, by using assumptions on the biological functions of specific genetic markers in choosing which marker is likely to interact with a specific environment in predicting a specific outcome. Thus, complementary to an inductive strategy—in looking for underlying mechanisms after a cG × E interaction has been robustly identified, as proposed by Dodge (2009)—we propose a deductive strategy.

### Toward a Theoretical Framework: Three Possible Underlying Mechanisms

**Fig. 1:**
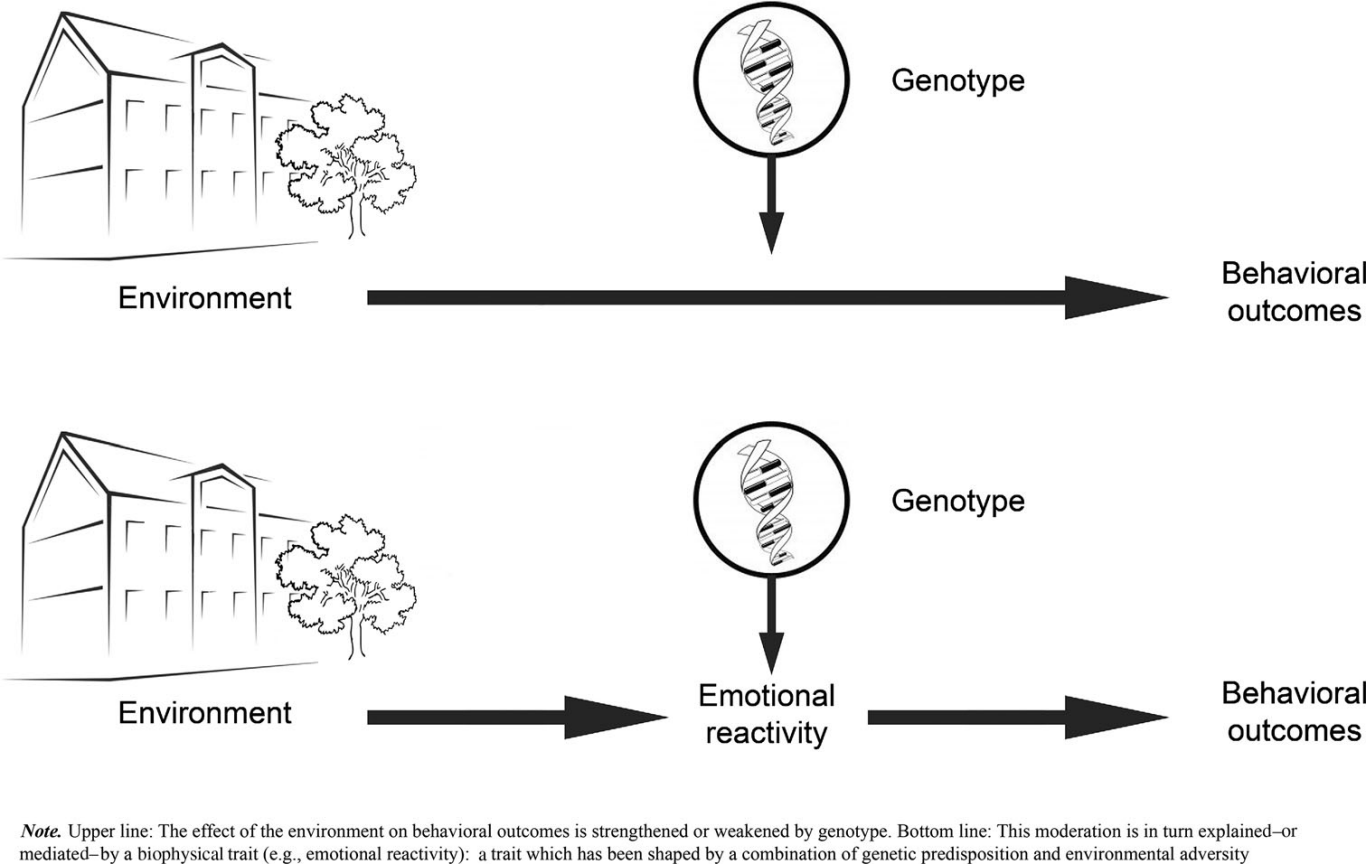
Conceptual mode of gene–environment interactions and underlying mechanisms

We are not the first to stress the importance of theories about mechanisms underlying cG × E (e.g., Reiss and Leve 2007; Rutter et al. 2006; Salvatore and Dick 2015), but to date only few scholars have elaborated on specific hypotheses on such mechanisms (see Davies and Cicchetti 2013; Dodge 2009; Calkins et al. 2013). Here, we put forward three possible, non-exclusive, explanatory mechanisms underlying G × E in the development of externalizing behaviors. These mechanisms concern serotonin-related emotional reactivity, dopamine-related reward sensitivity, and serotonin-related punishment sensitivity, and are based on associations previously established in the literature. Each mechanism takes the form of a mediated moderation model (i.e., the interaction between two variables affecting the mediator, which then affects a dependent variable, Morgan-Lopez and MacKinnon 2006; Muller et al. 2005), in which moderation by genotype is (partly) explained by a mediating process. The mediator is a partly heritable biopsychosocial function, which is shaped by countless previous interactions with the environment during early development (i.e., an entrained biopsychosocial trait, Dishion and Patterson 2006). Individual differences in these functions can both directly contribute to the development of externalizing behavior (e.g., as symptoms part of a diagnosis, such as impulsivity, irritability), as well as indirectly through the way children react to environmental adversity (e.g., heightened emotional reactivity to anger, lowered sensitivity to reward). In the case of G × E in the development of externalizing behavior, we assume that genetic make-up strengthens or weakens the effect of family adversity on externalizing problem behaviors, which is explained by individual differences in biopsychosocial functioning shaped by a combination of genetic predisposition and previous interactions with environmental adversity (see Fig. 1). Conform suggestions by Caspi and Moffitt (2006), for each mechanism we separately discuss evidence for the link between (1) externalizing behaviors (outcome) and the proposed mechanism, (2) genetics (G) and the proposed mechanism, and (3) family adversity (E) and the proposed mechanism.

### Emotional Reactivity

#### Externalizing Behavior and Emotional Reactivity

Individual differences in the form and intensity of reactions to emotional stimuli are at the core of temperament and personality research (Derryberry and Rothbart 1988) and are known risk factors in the development of externalizing behaviors (Scott and O’Connor 2012). Heightened emotional reactivity can function as an emotional liability that makes people more sensitive to negative emotional family environments (Sheese et al. 2009). Specifically, it may make people more likely to respond in affectively intense ways under stress. Indeed, in young children heightened emotional reactivity predicts temper tantrums (Giesbrecht et al. 2010). Research also shows that children’s irritable temperament is significantly associated with their angry reactivity to parental conflict and eventually to externalizing symptoms (Davies et al. 2012). Finally, differences in reactivity to emotional stimuli have been associated with different forms of externalizing psychopathology (e.g., De Wied et al. 2006).

#### Polymorphisms and Emotional Reactivity

Emotional reactivity is often referred to as an individual’s characteristic threshold, intensity, and duration of affective arousal (Rothbart and Derryberry 1981). In the neurobiological processing of emotional stimuli, the amygdala plays an important role (for a meta-analysis, see Costafreda et al. 2008). Differences in amygdala activity in reaction to emotional stimuli are associated with differences in imitation of emotional expressions, memory of emotional events, and social behavior (Decety 2010; Hare et al. 2008; Hariri and Holmes 2006; Pfeifer et al. 2008). The connectivity between the amygdala and the feedback circuit critical for emotion regulation is in turn shaped by variation in serotonin signaling (e.g., Pezawas et al. 2005).

Polymorphisms related to lower serotonin transport and uptake (specifically, the *5*-*HTTLPR* S-allele)—and therefore higher serotonin availability—have been associated with heightened amygdala activity in response to emotional stimuli (see for meta-analyses Munafò et al. 2008; Murphy et al. 2013). Although the exact mechanisms by which these polymorphisms are linked to amygdala response are unknown (Kobiella et al. 2011), one possibility is that these polymorphisms are related to diminished regulation and therefore higher and less stable availability of serotonin, causing higher increases in neuronal activity—and therefore higher arousal—during activation (for a review see Yildirim and Derksen 2013). These polymorphisms might thus, through biological translation and transcription pathways, ultimately contribute to a heightened neuronal reactivity to emotional stimuli. People carrying such polymorphisms might experience more intense and prolonged arousal when processing emotional stimuli than people without these polymorphisms. In the long run, this may lead to an up-regulated sensitivity for the effects of negative emotional stimuli and eventually lead to anger and irritability and the development of impulsive or reactive externalizing behavior (Miczek et al. 2002). Considering that this heightened emotional reactivity might manifest in irritable behavior in reaction to the environment, there is an important overlap with research on internalizing child behaviors. Irritability might therefore be, through emotional reactivity, an important underlying endophenotype to both oppositional behavior and child depression (Copeland et al. 2009; Stringaris et al. 2012).

#### Family Adversity and Emotional Reactivity

Environmental adversities such as harsh family emotional climates may also contribute to individual differences in emotional reactivity. The family is usually the first and most important context in which children learn how to recognize, interpret and manage other people’s emotions (Dunn et al. 1991; Dunn and Brown 1994). Negative parental emotional expressivity was found to be negatively related to children’s socioemotional competence and positively correlated with children’s externalizing problems (Isley et al. 1999). Children’s observations of marital conflict, for example, can lead to an increased sensitivity to anger cues (El-Sheikh et al. 1996). A recent meta-analysis found gray matter abnormalities in individuals exposed to childhood maltreatment, specifically in regions that are related to affect (Lim et al. 2014). Moreover, a whole-brain analysis showed that early family adversities differentially modify neural (i.e., amygdala and cortical) reactivity to emotional stimuli depending on *5*-*HTTLPR*-genotype (Walsh et al. 2012). It might therefore be that repeated exposure to negative family emotional climates increases neurological arousal to such emotional stimuli, in some children more than in others. Furthermore, such climates might cause changes in neuropsychological (e.g., emotion and behavior regulation), and attention processes (Davies et al. 2006). It has been shown that negative environmental cues might differentially alter activity in brain regions related to emotional processing: Amygdala and hippocampus activation at rest was correlated positively with life stress in carriers of the S-allele, but negatively in LL-genotypes (Canli and Lesch 2007).

In sum, the literature reviewed shows that: (1) high emotional reactivity is a risk factor for irritable, oppositional, and reactively aggressive behavior; (2) serotonin might be an important regulator of neuronal reactivity to emotional stimuli, with a low regulating serotonergic system (or high and unstable levels of serotonin) relating to high emotional reactivity, and (3) a negative emotional family climate might contribute to a heightened emotional reactivity. Thus, exposure to negative family emotional climates might increase neurological arousal by emotional stimuli, specifically in children with higher and less stable serotonin availability (e.g., *5*-*HTTLPR* S-allele; *MAOA* low-activity allele). In turn, children experiencing such dual risk might develop a heightened emotional reactivity. As a consequence, they might show more symptoms of angry/irritable mood (see symptom clusters of ODD in DSM-5, American Psychiatric Association 2013) and reactive aggression, compared to children growing up without this genetic and/or environmental risk, specifically when they are exposed to negative emotions and/or a negative family emotional climate (i.e., in line with frustration-aggression model; Berkowitz 1989). We know little about whether such a heightened emotional reactivity is specific to negative emotions, or might be a general heightened reactivity to both positive and negative emotions. In case of the latter, these children might benefit relatively more from interventions targeting the family emotional climate (e.g., teaching parents emotion-regulation strategies or targeting marital conflict). Also, because these children experience increased emotional arousal, they might specifically benefit from interventions targeting emotion regulation. Or, in case of heightened emotional reactivity to specifically negative emotions, from interventions using anger management techniques.

All hypotheses discussed in this paper are best tested using a triangulation of research strategies (Dick 2011). Longitudinal designs can be used to disentangle the prospective relationships between specific family/adversity (e.g., parental stress, warmth/harshness), the mechanisms (e.g., sensitivity to anger cues), and externalizing behavior (e.g., irritability and aggression), using specific measures for the environmental and behavioral variables. Marker for individual differences in dopaminergic or serotonergic regulation can serve as a moderator variable to test whether these relations are indeed stronger for some individuals than others (see Davies and Cicchetti 2013). In the case of the mechanism of emotional reactivity, the reviewed literature suggests that genetic markers that are specifically related to less efficient regulation of serotonin (i.e., high and unstable levels of serotonin) might be important measures of such differences in emotional stimuli. It might therefore be necessary to use multiple genetic markers, haplotypes or genetic pathways (e.g., using not only genetic variation in the serotonin transporter gene, but also including genes coding for the synthesis and reuptake of serotonin such as the *TPH1*, *HTR1A*, and *HTR2C)* as constructs of genetic moderation. Findings should be replicated in independent samples (see also Asherson and Price 2012). Simultaneously, more focused designs could be used to further investigate the mechanisms on a micro level (see also Howe et al. 2010). In case of the mechanism of emotional reactivity, experimental designs could be used to observe children’s emotional—and in turn behavioral—reactions to different emotional climates when interacting with their caregiver(s) (for an overview on experimental studies on cG × E, see Bakermans-Kranenburg and Van IJzendoorn 2015), for example, using facial electromyography (*f*EMG; e.g., Deschamps et al. 2012) or functional magnetic resonance imaging (fMRI, e.g., Canli et al. 2001) to measure the strength of children’s reaction to emotional stimuli.

### Reward Sensitivity

#### Externalizing Behavior and Reward Sensitivity

Reward insensitivity is related to externalizing behaviors through two different non-exclusive pathways. First, it is an important mechanism underlying differences in sensitivity to behavioral conditioning (Steinberg 2007). Insensitivity to reward might cause a lack of motivation to obtain ordinary or delayed rewards, resulting in an impaired social learning by stimulus-reward (Buckholtz et al. 2010). For example, aggressive boys with conduct problems have been found to show cardiac pre-ejection period (PEP) non-reactivity to monetary incentives, presumably indicating lowered reward sensitivity (Beauchaine et al. 2008). Second, low reward sensitivity may be related to excessive stimulation seeking (i.e., thrill- and sensation seeking) and egocentrically driven behavior (Quay 1988) as reaction to difficulties achieving a pleasant level of stimulation through regular sources (Matthys et al. 2013). Reward insensitivity might thus cause people to actively seek more powerful rewarding cues in their environment, ignoring negative cues, and not foreseeing long-term negative effects. In turn, this may be related to risk-taking and thrill-seeking behaviors and an increased risk for addiction (Robinson and Berridge 2008; Volkow et al. 1997).

#### Polymorphisms and Reward Sensitivity

Low reward sensitivity might be related to a blunted response to ordinary reward cues in the brain. The perceived value of reward is regulated by dopamine, in that dopamine activity has direct rewarding effects (Pessiglione et al. 2006; Schultz 2010). Specifically, the number of available dopamine receptors affects the level of stimulation by dopamine after it is released. Lower amount of receptors could therefore result in a reduced salience of positive environmental stimuli, making people less able to derive reward from ordinary, everyday activities (Buckholtz et al. 2010; Sevy et al. 2006). Furthermore, low uptake of dopamine might reduce effects of rewards on decisions (i.e., reward based learning), but also decrease valence of delayed rewards in both rodents and humans (for a review see Comings and Blum 2000). It might be that people carrying polymorphisms related to lower density of dopamine receptors in the brain are less aroused when dopamine is released in reaction to (anticipated) reward stimuli. Polymorphisms regulating dopamine availability and uptake predict interpersonal differences in neurological reactivity to reward cues (Dreher et al. 2009; Lancaster et al. 2012). Also, when looking at multilocus composite scores (which theoretically identify dopamine signaling capacity in reward regions), there is support for low reward activity in people with low dopamine signaling capacities (Stice et al. 2012). Additionally, it has been shown that children carrying a dopamine transporter genotype composite score related to lower transcriptional efficacy (and thus lower dopamine availability in the synapse) show more behavioral problems when mothers are unresponsive, than children carrying other genotypes, specifically because they show more behavioral disinhibition (Davies et al. 2015). Polymorphisms related to low dopamine activity might thus, through biological translation and transcription pathways, ultimately contribute to a lower sensitivity to ordinary or delayed rewards, causing a need for continuous, direct, and more salient reinforcers to control behavior (Kobayashi and Schultz 2008).

#### Family Adversity and Reward Sensitivity

Environments in which rewarding stimuli are less available (e.g., lack of positive parenting, abusive families, social or economic deprivation) might also contribute to differences in reward sensitivity. An animal model, for example, showed that monkeys deprived of parenting seemed less able to learn from rewards (Pryce et al. 2004). In humans, we see that growing up in families with a low social economic status may trigger a preference for immediate rewards, eventually predicting risky behaviors (Griskevicius et al. 2011). Also, people coping with addiction often have a history of abuse and maltreatment as children (Masten 2007). It might therefore be that the absence or scarcity of rewarding stimuli, in relation to behavior learning, lowers sensitivity to daily rewards, for some children more than for others. This might cause a need for more direct and larger rewards, and therefore impaired social learning by stimulus-reward, and active reward-seeking behaviors.

In sum, the reviewed literature shows that: (1) lowered reward sensitivity can be a risk factor for specifically non-compliant, antisocial, risky, thrill-seeking behaviors, (2) lower dopamine uptake after it is being released is associated with a reduced salience of positive stimuli, and (3) a preference for direct, large, and powerful rewards can be triggered by environments in which rewarding cues are less available. Thus, the absence or scarcity of rewarding stimuli might lower children’s sensitivity to daily rewards, specifically in children with low dopamine activity (e.g., *COMT* Val-allele, *MAOA* high-activity allele, *DRD4* 7-repeat allele, *DRD2* A1-allele, and *DAT* 10-repeat allele). In turn, children experiencing such dual risk might develop a low sensitivity to typical environmental reinforcers (i.e., experiencing them as less rewarding). As a consequence, they might show more non-compliant, risky, and thrill-seeking behavior, compared to children growing up without this genetic and/or environmental risk, specifically when the current environment does not offer them the necessary rewarding stimuli (e.g., specific praise and tangible rewards) or behavioral monitoring (i.e., short behavioral monitoring intervals). On the bright side, these children might respond specifically well to immediate versus postponed and strong versus weak reward. Therefore, using sufficient reward and interventions targeting reward-oriented parenting strategies, might be a very effective strategy to decrease problem behavior in these children. Also, if the emotional significance of the positive message of praise is less well processed, both verbal and nonverbal enthusiasm, accompanying praise and reward, would be particular relevant (Matthys et al. 2012). Furthermore, because these children are at risk for impulsive and risky behavior, these children might specifically benefit from interventions targeting behavioral control.

Experimental designs could be used to observe children’s behavioral reactions to parenting practices that focus on reward, and if these reactions are indeed stronger in some children than in others. For example, using a genetically informed experiment in which parents are assigned to different conditions and either instructed to use praise or small tangible rewards to condition a specific behavior or to use their usual approach (i.e., micro trial, Howe et al. 2010). The reviewed literature suggests that genetic markers which are specifically related to low dopamine activity (i.e., less efficient transport, low amount of receptors) might be important genetic markers of these individual differences in reward sensitivity, specifically in combination with family adversity. In older children, computer tasks can be used to measure differences in reward sensitivity between genetic subgroups (similar designs have been successfully used to study cG × E with different predictors see e.g., Gallardo-Pujol et al. 2013). Another promising approach might be embedding non-genetic biological markers, such as electroencephalography (i.e., EEG) measures, to genetic research on externalizing behavior. EEG has received increased attention over the last few years as potential biomarker for psychopathology and treatment response (for a review see Loo et al. 2015). Previous studies indeed indicate that resting-state brain activity (specifically theta activity) is related to reinforcement learning, risky decision taking, and might therefore be a marker for specifically reward, but not punishment, sensitivity (e.g., Massar et al. 2014).

### Punishment Sensitivity

#### Externalizing Behavior and Punishment Sensitivity

Individual differences in punishment sensitivity are mostly seen as differential responsiveness to fear conditioning (Eron 1997; Lykken 1957). Low punishment sensitivity may result in a lower concern about consequences of behavior. This may become manifest as disregard for aversive consequences of response choices (Fontaine 2006), in reward-driven behavior, and in difficulties changing one’s behavior in response to punishment cues (Carlson et al. 2012; Santesso et al. 2011). Low punishment sensitivity is associated with aggression in childhood and criminal behavior in adulthood (Gao et al. 2010a, b). Also, children with ODD are less likely than controls (and than children with ADHD) to change behavior after it was punished (Humphreys and Lee 2011; Matthys et al. 2004). Longitudinal research further found that children with low levels of temperamental fear are less receptive to discipline techniques that are based upon punishment (Kochanska 1997). Research indicates that specific aspects of punishment insensitivity, namely low anxiety and sensitivity to aversive stimuli, and high reward dominance, are evident in clinical samples of adolescents (for an overview see Dadds and Salmon 2003).

#### Polymorphisms and Punishment Sensitivity

Low punishment sensitivity is a largely heritable factor consisting of a diverse but overlapping set of propensities including low arousal to aversive stimuli, fearlessness, and poor avoidance learning (Dadds and Salmon 2003). Several functional neuroimaging studies have shown that the amygdala and related structures are important in processing cues related to threat and fear (for a review see Davis and Whalen 2001). People high on externalizing behaviors possibly show reduced amygdala reactivity to—particularly negative—emotional stimuli (Blair 2008; Sterzer et al. 2005). This failure to be aroused by and learn from stressful stimuli or punishment may predispose individuals to deficient conscience development and poorly socialized behaviors (Kochanska et al. 2007).

Serotonin might act as a motivational opponent to dopamine (Daw et al. 2002) by modulating the impact of punishment-related (rather than reward-related) signals on learning and emotion (Cools et al. 2008). Children and adolescents showing high levels of externalizing behaviors seem to show altered serotonergic functioning (for a review see Matthys et al. 2013). In contrast to high and less stable serotonin availability underlying a heightened emotional reactivity, low and stable serotonin availability might underlie reduced punishment sensitivity. Indeed, *5*-*HTTLPR* L-allele carriers exhibit low amygdala activity in reaction to emotional stimuli (as low as 3 % compared to 28 % in S-allele carriers; see Munafò et al. 2008). Also, *5*-*HTTLPR* L-allele carriers showed impairments in avoidance learning and show overall lower fear responses, compared to S-carriers (Finger et al. 2006; Brocke et al. 2006). Low and stable serotonin availability possibly lowers the intensity and duration of emotional arousal, lowering stress sensitivity in reaction to emotional stimuli (for a review see Yildirim and Derksen 2013). These polymorphisms might thus, through biological translation and transcription pathways, ultimately contribute to lower arousal by aversive stimuli, and therefore insensitivity to punishing cues, such as anger and distress in others, as well as to aversive consequences following externalizing behavior. Given this possible pathway, it is not surprising that reduced punishment sensitivity has been suggested as a key factor in psychopathy (Frick and Ellis 1999). Parallels exist between research on psychopathy and the described literature on punishment sensitivity, showing similarities in behavioral traits, polymorphisms, brain functioning, and neuropsychological indicators (for reviews see Glenn 2011; Yildirim and Derksen 2013).

#### Family Adversity and Punishment Sensitivity

Although punishment sensitivity is mostly seen as a child factor it is also partly dependent on environmental factors (for an overview see Dadds and Salmon 2003). As early as the 1950s, it was found that animals raised in deprived environments show less intense reactions to pain stimuli (Nissen et al. 1951) and take longer to learn avoidance of painful stimuli (Melzack and Scott 1957). In humans, maltreated children show a disregard for risk of punishment when responding in a reward-oriented task (Guyer et al. 2006). It might therefore be that in some children, more than in others, consistent harsh punishment or punishment mixed with reward reduces their reactivity to punishment. This makes it difficult to further socialize these children through strategies that rely on motivation to avoid punishment instead of to obtain reward.

In sum, the reviewed literature shows that (1) low punishment sensitivity is a risk for—specifically proactive—externalizing behaviors, (2) serotonin acts as a motivational opponent to dopamine in that it regulates the impact of punishment-related signals in which low/stable levels of serotonin are related to low punishment sensitivity, and (3) harsh or unpredictable family environments can increase insensitivity to punishment and therefore increase reward-oriented behaviors. Thus, children experiencing maltreatment, harsh punishment, or punishment mixed with reward might develop a blunted reactivity to negative emotional arousal and punishment, specifically when they have low and stable levels of serotonin availability (e.g., the *5*-*HTTLPR* L-allele or *MAOA* high-activity allele). In turn, children experiencing such dual risk might be less sensitive to punishment-oriented strategies and will therefore show more antisocial behavior compared to children growing up without this genetic and/or environmental risk, specifically when the current environment relies on harsh and/or unpredictable punishment for socialization. Eventually, this low reactivity and poor conditionality through punishment might induce proactive, instrumental, and maybe even predatory antisocial behavior later in adulthood, forming a risk factor for proactive agression, antisocial personalities and psychopathic traits. Also, these children might be at risk for escalating cycles of punishment, as milder forms of punishment may be less effective and parents might get frustrated. On the bright side, interventions focusing on relabeling inappropriate behaviors into positive opposites, and using praise and token economy to positively reinforce the appropriate behaviors, might be especially effective for this group of children (see also Matthys et al. 2012). Also, because these children might be at risk for deficiencies in emotion recognition, empathy, and perspective taking, they might specifically benefit from cognitive behavioral treatment.

Randomized controlled trials using a parenting intervention to reduce punishment-oriented strategies can be used to test this punishment sensitivity hypothesis. Such RCT’s could be used to explore whether such interventions have different effects on externalizing behavior between different genetic subgroups (similar designs have been previously used see Bakermans-Kranenburg and Van IJzendoorn 2015). The reviewed literature suggests that genetic markers which are specifically related to low and stable levels of serotonin availability (i.e., low levels of synthesis, high levels of reuptake and degradation) might be important genetic markers of these individual differences in punishment sensitivity, specifically in combination with family adversity. Within more focused experimental research, different physiological measures could be used to assess punishment sensitivity. For example, a previous study demonstrated the value of startle reactivity measures for differentiating between mechanisms underlying the development of different externalizing phenotypes (Fanti et al. 2015). Since the amygdala plays a role in punishment decision-making (e.g., Treadway et al. 2014), fMRI might be a promising additional measure of individual differences in punishment sensitivity (e.g., Gregory et al. 2015).

## General Discussion

In part one of our paper, we systematically reviewed 53 papers on cG × E in externalizing behaviors, showing many significant, but also contrasting, findings. Large variations in methodologies (e.g., sample size and composition) and differences in—the specificity of—conceptualizations of both family adversity and externalizing behaviors make it difficult to integrate these findings and make cross-study comparisons. This essentially means that many cG × E studies are oftentimes built on earlier findings of not necessarily comparable studies, and thus not serving to create a coherent base of literature. One way to create more comparable results, and draw solid conclusions, is by forming a priori hypotheses on how specific family adversity factors interacting with specific polymorphisms in specific samples predict specific behavior. Researchers should be able to justify why they are studying these specific genes, this environmental risk, this behavioral outcome, and why they measured it with these specific instruments (see also Dick et al. 2015). Therefore, it is imperative to move toward a deductive strategy by forming theories on the interaction between genes and environment, which not only stem from cG × E research, but also from literature on behavior development, genetics, and neurobiology. The added bonus of working with a sound theoretical framework is that it might help to diminish publication bias by enabling us to confirm or reject theory-based hypotheses and thus increase the relevance of “null findings” (i.e., nonsignificant interactions).

In part two of our paper, we therefore tried to contribute to the literature by proposing three hypotheses on non-exclusive mechanisms underlying G × E in externalizing behavior. Concrete evidence emerged from the literature review for the mechanisms of emotional reactivity, reward sensitivity, and punishment sensitivity. These mechanisms most likely underlie specific traits rather than diagnoses or complex behavioral clusters. Because of high comorbidity and overlap in symptomology and etiology within externalizing behavior, both equifinality and multi-finality is to be expected. Different genetic and neuropsychological pathways might contribute to the same diagnosis (i.e., equifinality). For example, CD is associated with cG × E including the *MAOA* (Foley et al. 2004), as well as the *DAT1* and *5*-*HTTLPR* (Sonuga-Barke et al. 2009). At the same time, a single polymorphism may contribute to different behavior clusters (i.e., multi-finality). For example, the *5*-*HTTLPR* S-allele predicts heightened emotional reactivity, which includes irritability, a core symptom of both depression and ODD in children (Copeland et al. 2009). It could thus be that serotonin-related heightened emotional reactivity underlies both disorders through the same distinct genetic underpinnings for this trait (Stringaris et al. 2012). The term “risk” allele is therefore questionable in the context of cG × E. Indeed, some alleles have even been shown to “protect” from certain adversities and have been related to children’s heightened susceptibility to parenting interventions (Bakermans-Kranenburg et al. 2008). Although this paper focused on differential negative effects of family adversities (i.e., dual risk), the described mechanisms might work both ways, in such that the same mechanisms might lead to differential pro-social behavioral outcomes under environmental enrichment (i.e., differential susceptibility, Belsky 2005). Also, it is important to note that the proposed mechanisms should be seen as possible candidates, but neither as comprehensive frameworks in explaining externalizing behaviors through cG × E nor as providing a quick fix to overcome all growing pains of this field. Specifically, the role of executive functions, meta-cognitive functions, and morality in these mechanisms needs further elaboration. Deviances in the processing of reward and punishment cues may, for example, affect cognitive functions such as decision-making (Matthys et al. 2013). A recent study found that emotion regulation through cognitive reappraisal serves as a buffer in the association between *5*-*HTTLPR*, environmental stressors, and psychopathology (Ford et al. 2014). Also, to narrow the scope of our literature overview, our review focuses solely on family adversity. However, the effects of specific family adversity might change over time (Choe et al. 2014; Reiss et al. 2013; Windhorst et al. 2015). Specifically, environmental adversity measures outside the family might become increasingly important when children grow older. Indeed, a recent study found differential associations of a polygenetic score with adolescent externalizing behavior when including different environmental adversities, with stronger effects for peer substance use than for parental monitoring (Salvatore et al. 2014). Moreover, environmental risk factors are not independent factors, and together form the broader environmental context children grow up in (see for a review of this issue Boardman et al. 2013). Another limitation of our review paper is that the interplay between genes and the environment might be too complex to be explained through moderation (or G × E) alone. We need to take into account that genes might, for example, also control the exposure to certain environments (i.e., gene–environment correlation, see Plomin and Simpson 2013). For example, children low on reward sensitivity might actively seek out risky environments and children low on punishment sensitivity might evoke harsh parenting behavior. Furthermore, current knowledge on functional expressions of the polymorphisms in the human brain is limited (Balciuniene et al. 2002). Multiple inherited DNA elements can influence transcription and expression of a protein (see also Rutter 2007). In the search of underlying mechanisms, it might therefore be important to relate these mechanisms to one or more functional genetic pathways or haplotypes (see also Plomin and Simpson 2013). More recent studies on the functional effects of the *MAOA*, for example, showed no significant association of a single polymorphism with expression levels or enzyme activity in the human brain, but did find such associations with a haplotype (Balciuniene et al. 2002). Although not reviewed in this paper, some of the reviewed studies found interactions among multiple candidate genes (often with very different functions). For example, Simons et al. (2011) found an interaction between the *DRD4* (coding for dopamine receptors), *5*-*HTTLPR* (coding for serotonin transporters) polymorphisms, and social conditions in predicting aggression. It is difficult to address functional mechanisms for such polygenic effects of functionally diverse polymorphisms. One possibility is that such polygenic effects indicate cumulative genetic vulnerability or a complex interplay of genes on regulation of different neurotransmitters (e.g., they might bring about a certain balance in neurotransmitter activity). There are, however, also examples of cumulative effect of functionally related groups of genes. For example, Stephens et al. (2012) found a direct association between multiple SNP’s in the *CHRNA5/CHRNA3/CHRNB4* (i.e., neuronal nicotinic acetylcholine receptor) gene cluster and externalizing behavior.

Another important issue for future research is that, specifically in early development, differential developmental outcomes might be caused by environmental influences that alter the functional activity of genes without altering the sequence (i.e., epigenetics, see Roth 2013). DNA methylation, for example, mediates the relation between a polymorphism and developmental outcomes by changing the expression of the gene (Van IJzendoorn et al. 2011). Candidate gene approaches have been criticized for their rather naïve view on the biological function of single genes (e.g., Szyf and Bick 2013), and technological progress enables us to use more advanced strategies to study the role of genetics in externalizing behavior (e.g., genome-wide association studies (GWAS), haplotype analyses, genetic pathways, epigenetics). These approaches can deepen and extend our knowledge on the role of genetics in the development of externalizing behavior. However, it is important to note that these strategies do not necessarily yield more consistent findings (Aebi et al. 2015; Chabris et al. 2013; Neale et al. 2010). For example, results of a GWAS study on CD by Dick et al. (2011) showed no overlap with findings of a GWAS study on CD by Anney et al. (2008). Also, due to a lack of knowledge on the functions of many “new” genes, it is sometimes not possible to interpret findings and describe functional involvement of these genes in the development of specific behavior (e.g., Dick et al. 2011; Pappa et al. 2015). Moreover, the same recommendations on using theory-based a priori hypotheses apply to studies using such more advanced methods. Studies on the function, expression, and effects of single polymorphism can possibly be seen as “links in a chain,” functioning as important stepping-stones for moving us forward. Polymorphisms having a functional impact on gene expression can function as markers for more complex processes underlying individual differences in reaction to family adversity, and therefore provide us with clues on possible underlying mechanisms. Such a “reversed endophenotype” (see also Loo et al. 2015) approach could also help us in the search for less intrusive markers for differential susceptibility to specific environmental adversity, and in turn give us clues for research and intervention strategies.

## Conclusion

Findings on cG × E in externalizing behaviors are heterogeneous. However, large methodological differences between studies make it difficult to integrate findings and draw solid conclusions on the role of cG × E in externalizing behavior. Hypotheses on underlying mechanisms of cG × E can serve as a conceptual framework for gaining a deeper understanding of these interactions, specifying our research strategies accordingly, and substantiate the findings reported so far. Hypotheses that derive from these frameworks should be tested, using a multidisciplinary (i.e., Play nice in the sandbox, Dick 2011) triangulation of research strategies (Overbeek et al. 2012). Although this review raised a series of important issues in the field of cG × E that need to be resolved, it also bears an optimistic message: The literature holds many clues on possible mechanisms. Insight in the underlying mechanisms can possibly help us interpret the intriguing, but inconsistent, findings on cG × E and enhance their empirical and clinical implications. It can point us in the direction of differential pathways leading to externalizing behaviors. And though not directly implementable, it can provide us with more insight in individual differences in the development of externalizing behavior. Eventually, knowledge on specific cG × E and their mechanisms might, for example, enable us to better predict which children are specifically vulnerable in which developmental period and target them using personalized interventions, not only in terms of clinical focus (i.e., based on specific mechanisms at work) (Matthys et al. 2012), but also in terms of intensity and duration (i.e., based on differences in susceptibility).
